# The glymphatic pathway in the optic nerve: did astronauts already reveal signs of its existence?

**DOI:** 10.1038/s41526-021-00142-y

**Published:** 2021-04-19

**Authors:** Peter Wostyn, Charles Robert Gibson, Thomas H. Mader

**Affiliations:** 1 Department of Psychiatry, PC Sint-Amandus, Beernem, Belgium; 2 Coastal Eye Associates, Webster, TX USA; 3 KBR, NASA Space medicine Operations Division, Houston, TX USA; 4 Colonel, US Army (retired), Moab, UT USA

**Keywords:** Anatomy, Optic nerve diseases

**Arising from** Rohr J. J. et al. *npj Microgravity* 10.1038/s41526-020-00119-3 (2020)

The recent study by Rohr et al.^1^ that was published in *npj Microgravity* aimed to provide a quantitative analysis of optic nerve and optic nerve sheath (ONS) cross-sectional areas, and optic nerve deviation, an indication of tortuosity, before and after long-duration spaceflight (LDSF). High-resolution, T1-weighted sagittal, and T2-weighted coronal magnetic resonance imaging (MRI) scans were collected from ten astronauts undergoing ∼6-month missions on the International Space Station (ISS). Each astronaut underwent MRI scanning before flying to the ISS and at five recovery time points after return to Earth (approximately 3, 30, 90, 180, and 360 days after landing). On average, optic nerve cross-sectional areas tended to decrease in their cohort immediately post flight. However, only the 30 days postflight time point showed a statistical difference from preflight, with an average reduction in optic nerve cross-sectional area of −0.89 mm^2^. The exact reason for this optic nerve area reduction is unclear. As noted by the authors, although increased cerebrospinal fluid (CSF) pressure surrounding the optic nerve can lead to axoplasmic flow stasis and resultant optic disc edema, it is unclear how these changes may specifically impact the optic nerve morphology during and after LDSF. They also documented no significant increase in the ONS diameter following LDSF except in one astronaut with unilateral grade 1 optic disc edema. This suggests that, in nine of the ten astronauts, intracranial pressure was not pathologically elevated immediately after spaceflight. The authors appropriately mention that the ONS in astronauts may demonstrate hysteresis such that exposure to ONS pressure beyond a certain point may result in failure of the sheath to regress to normal following spaceflight and change its response to subsequent pressure changes. It would be interesting to know how many of the ten astronauts they report had previous spaceflights prior to their MRI study that might have impacted their ONS response to repeat extended microgravity exposure.

The authors suggest that a possible explanation for the decrease in optic nerve cross-sectional area could be the role of changes in the optic nerve vasculature^1^. They discuss a retrospective MR study by Kramer et al. published in 2012^2^. In that study, the authors observed a central area of T2 hyperintensity in the optic nerve in 26 of the 27 astronauts (96%) with a history of microgravity exposure^2^. As discussed below, we propose that this T2 hyperintensity in the optic nerve reflects a glymphatic pathway and that altered dynamics of the optic nerve glymphatic circulation may contribute to the reduction in the optic nerve cross-sectional area observed after spaceflight.

We raise the question of whether the previously reported T2 hyperintensity in the optic nerve of astronauts^2^ already reflected signs of the existence of glymphatic flow of CSF into the optic nerve. The “glymphatic system” is a recently discovered brain-wide clearance system in which CSF enters the brain via periarterial channels and exchanges with interstitial fluid^3^. This fluid is then removed from the brain along perivenous pathways and eventually cleared via the cervical lymphatic vasculature^3^. CSF within the subarachnoid space (SAS) is gradually forced into the Virchow-Robin spaces by the combined effects of arterial pulsatility, respiration, slow vasomotion, and CSF pressure gradients^4,5^. Thereafter, CSF passes into the dense brain parenchyma via perivascular astrocytic aquaporin-4 (AQP4) water channels^4^.

The optic nerve, a white matter tract of the CNS consisting of afferent axons of the retinal ganglion cells, is also surrounded by CSF within the orbital SAS^6^. Recently, Mathieu et al.^6^ provided evidence to support the existence of a glymphatic pathway in the optic nerve following tracer injection into the CSF of live mice. Their findings indicated that CSF enters the optic nerve via spaces surrounding blood vessels, bordered by AQP4-positive astrocytic endfeet. We recently hypothesized that the forcing of perioptic CSF into the perivascular spaces surrounding the central retinal artery, as a result of long-standing microgravity fluid shifts, may partly explain the optic disc edema observed in astronauts during LDSF^7^. It is important to note that individual variations in ONS anatomy and compliance may impact the end result of this process^7^. The MR study of astronauts by Kramer et al.^2^ seems to support the possibility that the optic nerve communicates directly with its SAS. In cases with an ONS kink, the authors documented a significant increase in the diameter of the central area of T2 hyperintensity in the mid optic nerve^2^. They also noted that the kink produced an increase in ONS diameter suggestive of a localized rise in CSF pressure. The authors hypothesized that a kink may distort the complex system of arachnoid trabeculae, pillars, and septa within the perineural SAS^2^. This ONS deformation could lead to a restriction of anterograde CSF flow and a resultant pressure gradient^2^. Furthermore, this process could result in the proximal congestion of the perivascular space of the optic nerve via communication with the perineural SAS^2^. In another study by Riascos et al.^8^ of 21 astronauts, MR examination documented a central T2 hypointensity in the epicenter of the previously described T2 hyperintensity in the optic nerve in all those examined, including the two astronauts not yet exposed to microgravity, suggesting the T2 hypointensity may have been created by a central retinal vessel flow void artifact.

Considering the above, we believe that the reduction in optic nerve cross-sectional area observed after spaceflight may result, at least partly, from altered dynamics of the optic nerve glymphatic circulation. Indeed, while prolonged exposure to microgravity may predispose to an overload in the periarterial inflow of CSF into the optic nerve^7^, we hypothesize that in those astronauts without pathologically elevated postflight CSF pressures within the ONS, the rapid fluid redistribution upon return to Earth, which consequently may lead to reduced CSF volume and pressure within the ONS, may decrease CSF inflow along the optic nerve periarterial glymphatic spaces. As a result, after landing on Earth, these periarterial spaces may dramatically reduce and even disappear, at least partly contributing to the observed decrease in optic nerve cross-sectional area. In order to further explore and validate this hypothesis, it would be interesting to measure changes in the diameter of the central area of T2 hyperintensity in the optic nerve before, as soon as possible after, and at several more recovery time points following spaceflight.
